# The Fourth Bioelectronic Medicine Summit “Technology Targeting Molecular Mechanisms”: current progress, challenges, and charting the future

**DOI:** 10.1186/s42234-021-00068-6

**Published:** 2021-05-24

**Authors:** Timir Datta-Chaudhuri, Theodoros Zanos, Eric H. Chang, Peder S. Olofsson, Stephan Bickel, Chad Bouton, Daniel Grande, Loren Rieth, Cynthia Aranow, Ona Bloom, Ashesh D. Mehta, Gene Civillico, Molly M. Stevens, Eric Głowacki, Christopher Bettinger, Martin Schüettler, Chris Puleo, Robert Rennaker, Saroj Mohanta, Daniela Carnevale, Silvia V. Conde, Bruno Bonaz, David Chernoff, Suraj Kapa, Magnus Berggren, Kip Ludwig, Stavros Zanos, Larry Miller, Doug Weber, Daniel Yoshor, Lawrence Steinman, Sangeeta S. Chavan, Valentin A. Pavlov, Yousef Al-Abed, Kevin J. Tracey

**Affiliations:** 1 The Feinstein Institutes for Medical Research, Northwell Health, Manhasset, NY USA; 2 Karolinska Institute, Solna, Sweden; 3 University of Utah, Salt Lake City, UT USA; 4 National Institutes of Health, Bethesda, MD USA; 5 Imperial College of London, London, UK; 6 Linköping University, Linköping, Sweden; 7 Carnegie Mellon University, Pittsburgh, PA USA; 8 CorTec GmbH, Freiburg im Breisgau, Germany; 9 General Electric, Niskayuna, NY, USA; 10 University of Texas at Dallas, Richardson, TX USA; 11 Institute for Cardiovascular Prevention, Ludwig-Maximilians-University, Munich, Germany; 12 Sapienza University of Rome, Rome, Italy; 13 IRCCS Neuromed, Pozzilli, Italy; 14 CEDOC, Nova Medical School, Faculdade de Ciências Médicas, Lisbon, Portugal; 15 University of Grenoble Alpes, INSERM, Grenoble, France; 16 SetPoint Medical, Santa Clarita, CA, USA; 17 Mayo Clinic, Rochester, MN, USA; 18 University of Wisconsin, Madison, WI USA; 19 University of Pennsylvania, Philadelphia, PA USA; 20 Stanford University, Stanford, CA USA

**Keywords:** Bioelectronic medicine, Summit, Vagus nerve stimulation, Feinstein Institutes for Medical Research, Materials science, Electronics, Devices, Preclinical research, Clinical trials, Neural circuits

## Abstract

There is a broad and growing interest in Bioelectronic Medicine, a dynamic field that continues to generate new approaches in disease treatment. The fourth bioelectronic medicine summit “Technology targeting molecular mechanisms” took place on September 23 and 24, 2020. This virtual meeting was hosted by the Feinstein Institutes for Medical Research, Northwell Health. The summit called international attention to Bioelectronic Medicine as a platform for new developments in science, technology, and healthcare. The meeting was an arena for exchanging new ideas and seeding potential collaborations involving teams in academia and industry. The summit provided a forum for leaders in the field to discuss current progress, challenges, and future developments in Bioelectronic Medicine. The main topics discussed at the summit are outlined here.

## Introduction

Bioelectronic Medicine (BEM) is a rapidly developing and evolving field that explores advanced applications of neuromodulation in disease treatment (Pavlov and Tracey 2019). Preclinical research in BEM continues to generate new insights into neural regulation of physiological functions and technological developments in bioelectronics. These advances provide a rationale for new clinical trials that evaluate novel bioelectronic treatment approaches. There are many diseases and conditions for which conventional pharmacological treatments do not provide sufficient or any benefit and BEM has started to provide viable alternatives. Prominent examples are rheumatoid arthritis (RA), inflammatory bowel disease (IBD) and other GI disorders, cardiovascular disease, spinal cord injury and paralysis, neurodegenerative diseases, post-stroke neurological deterioration, and many others.

It is critically important that scientists, clinicians, engineers, and representatives of industry and policymakers with common interests in BEM meet to discuss current progress and challenges in the field. The fourth bioelectronic medicine summit “Technology Targeting Molecular Mechanisms” resonated the importance of better understanding of neural regulation at a molecular level that can be interrogated by advanced electronics in targeted disease treatments. The virtual meeting was hosted by The Feinstein Institutes, Northwell Health and sponsored by CorTec GmbH and United Therapeutics. An organizing committee, including Octavia Davis, Meredith Burcyk, Sangeeta Chavan, Valentin Pavlov, Yousef Al-Abed, and Kevin Tracey aimed at providing a forum for discussing various important aspects of BEM with relevance to the growing and diverse BEM community. The online meeting was an arena for engaging discussions between speakers, session chairs, and participants from the audience during the sessions and poster presentations. The abstracts of the posters presented at the summit were recently published (Abstracts from the fourth bioelectronic medicine summit: technology targeting molecular mechanisms 2020). An important event during the summit was dedicated to hearing the voice of the patient in disorders such as IBD and RA, in which BEM approaches have started to be successfully explored. This session was organized and chaired by Kelly Owens from The Feinstein Institutes. The highlight of the meeting was the keynote address by Larry Steinman from Stanford Medicine. Dr. Steinman discussed findings published during the last 20 years in Nature, Science, PNAS and other high profile journals about the beneficial effects of amyloid structures in multiple sclerosis and other diseases and how they engage the alpha7 nicotinic acetylcholine receptor into a regulatory anti-inflammatory mechanism. The alpha7 nicotinic acetylcholine receptor is a key signaling molecule in the vagus nerve-based inflammatory reflex, which is a major focus of therapeutic exploration in bioelectronic medicine. Dr. Steinman spoke about the specific role of aB-Crystallin as a guardian molecule in multiple sclerosis and a variety of other disorders, including stroke and ischemic optic neuropathy. He also elaborated on the molecular mechanisms of the anti-inflammatory protective effects of aB-Crystalline and its hexapeptide components that importantly involve signaling through the alpha7 nicotinic acetylcholine receptor expressed on immune cells. These findings indicate exciting new possibilities for applying anti-inflammatory BEM approaches in the treatment of different diseases. In the following sections, we outline the main topics of BEM research and clinical applications discussed at the summit in appropriately structured sessions with experts in the field as chairs and speakers.

## Materials science & electronics

The session was chaired by Chad Bouton and Loren Rieth from the Feinstein Institutes. BEM critically depends on approaches that enable safe and effective recording, stimulation, and/or modulation of the nervous system. While significant advances have been already achieved, there are some challenges and limitations. A key limitation to this endeavor is related to optimizing the signal-to-noise ratio (SNR) in both stimulation and recording. For stimulation, this concept is highlighted by the need to selectively stimulate the nervous system to achieve therapeutic benefit (signal), without activating undesired responses and side-effects (noise). For recording neural signals, measuring neural signals from specific fibers or regions (signal) is dramatically complicated by noise from other neurons, muscles, and anthropomorphic sources (noise). These remain challenges, despite tremendous progress in the materials, electronics, and their associated architectures, even for acute and non-survival pre-clinical studies. These challenges are intensified for chronic (> 30 days) and long-term (> 1 year) technologies, where significant decreases in the SNR for microelectrode technologies are the norm, not the exception.

The two broad approaches to improve this can be characterized as top-down and bottom-up approaches. Top-down approaches are focused on scaling down the size and improving the selectivity of established technologies. Examples include increasing the number of electrodes for traditional vagus nerve cuffs, multi-contact DBS electrodes, and cochlear electrodes with more stimulation sites. Bottom-up approaches explore opportunities to use new materials, electronics, packaging, and integration to improve the SNR of stimulation, recording, and modulation of the nerves, and ability to do so over the long term. Examples include NeuroPixels probes, research efforts by Viventi and Shepard, and devices from Iota. Each approach has benefits and disadvantages that have been reviewed.

At the meeting, the discussion largely focused on bottom-up approaches to improve the SNR, safety, and efficacy of BEM technologies, as well as overarching perspectives from the NIH on past successes and future directions for BEM research. The panel included luminaries and leaders in the field, as well as exciting new efforts from a new generation. The panel included Molly Stevens from Imperial College London who spoke about the potential to facilitate the safe and effective transfer of charge from electronics to neural substrates, with the incredibly broad ability to tune this process through the diversity of backbones (Mawad et al. 2016; Higgins et al. 2020; Ritzau-Reid et al. 2020; Spicer et al. 2017) and moieties at her disposal. Prof. Christopher Bettinger from Carnegie-Mellon University presented his work on new polymeric materials that are compatible with microfabrication, and can achieve reliable adhesion to cellular (neural) substrates (Golabchi et al. 2019). Achieving and maintain intimate contact between electrodes and the nervous system, while avoiding trauma from surgical placement, tethering forces, or compressing vasculature is a long-standing challenges to the field. Eric Daniel Głowacki from the Laboratory of Organic Electronics at Linköping University presented on his innovative use of organic photovoltaic materials as a label-free mechanism for peripheral nerve stimulation (Ðerek et al. 2020). Stability of organic electronics has been a significant challenge, highlighting the importance of his ability to achieve functional lifetimes for devices in-vivo. Eugene Civillico is the program manager for the NIH program of Stimulating Peripheral Activity to Relieve Conditions (SPARC), the primary program within the NIH dedicated to BEM. He provided a comprehensive overview of past and current successes, described an innovative and interactive pantheon of online resources to dramatically improve data sharing and analysis, and highlighted an open Request for Information being used by the SPARC team to take input on future directions.

The engaging discussion made clear the strong need for tools both for basic research, and as part of the next generation of devices to instantiate and broaden the range of bioelectronic medicine applications. At the level of basic science, it is clear that there is still a significant need to better understand the neural circuits and reflexes needed to maintain homeostasis and health, and how they can be modulated to treat disease. Innovations from this hypothesis and discovery driven research then require translation to achieve clinical impact in our ability to treat disease. This involves hardening the technologies into safe and effective devices that can receive regulatory approval and be profitably marketed to make the world healthier.

## Bioelectronic devices in preclinical research

This session was chaired by Timir Datta-Chaudhuri and Dan Grande from the Feinstein Institutes. Preclinical research in BEM is of fundamental importance for providing mechanistic insights and evaluating therapeutic strategies in disease treatments that can be further developed in the clinic. Stavros Zanos from The Feinstein Institutes spoke about current progress and problems with current clinical vagus nerve stimulation (VNS) therapies that rely on engaging vagal fibers in a relatively uncontrolled manner, which sometimes results in sub-therapeutic doses to targeted organs and side-effects from non-targeted organs. His research efforts aimed to quantify the engagement of vagal fibers and end-organs and presented strategies to target sub-populations of fibers on an individual subject basis. He presented data from experiments carried out in different animal models of VNS with clinically preferred, non-penetrating cuff electrodes. Such methods for “targeted vagal neuromodulation” were shown to be useful for using VNS to treat diseases in which hemodynamic state can easily be compromised, like heart failure and pulmonary hypertension. Dr. Zanos elaborated on the mechanisms, fiber types and neural circuits by which the autonomic nervous system informs the brain about the status of peripheral organs and systems and exerts control over them. He highlighted approaches to map the peripheral and central neural circuits responsible for these functions and to track how these circuits are altered by disease. His methods include physiological (Chang 2020), electrophysiological (Chang 2019, Levy 2019), anatomical (Toth 2020), computational (Lin 2020), and closed-loop (Zanos 2019, Zanos 2018) approaches which are used to understand neural activity related to autonomic function in the nerves themselves, the ganglia and the brain and how nerve stimulation affects the brain and the organs to which the nerves project. It is clear that improved understanding of the fiber engagement is critical towards the goal of developing selective stimulation strategies. VNS plays a central role in the ongoing development of new BEM therapies, yet the reasons that the vagus nerve is an appealing target are the same reasons why VNS can lead to off-target effects. The cervical vagus nerve presents an opportunity for stimulation before the nerve branches out several peripheral organs; it is easy to access surgically and its relatively large size allows the use of easy to manufacture electrodes. However, because the cervical region is a trunk location, targeting specific fibers remains a challenge. The literature is rich with methods for selective targeting of nerves based on fiber morphology and stimulation parameters, but these methods have not been translated into clinical embodiments, e.g. (Ahmed 2020). This type of fundamental research, starting with small animal models and moving to larger to representative models before finally moving on to clinical translation is key to the development of target specific bioelectronics therapies.

Martin Schuettler from CorTec presented the work being done at his company on a chronically implantable brain-computer interface called “Brain Interchange” for bi-directional communication with the brain. A detailed description of the system consists of various components: cortical grid electrodes, implantable electronics protected by ceramic hermetic package and body-external transceiver that communicates wirelessly with the implant and also wirelessly supplies the implant with energy via an inductive link. The system streams neural data from 32 electrode contacts to a computer where the data is processed and stored. Based on the incoming neural data, algorithms on the computer can take decisions on therapeutic actions, e.g. sending a stimulation command wirelessly to the implant which then delivers electrical pulses via the implanted electrodes to the brain, modulating brain activity. This closed-loop system can be used for patient-individual and situation-specific brain therapy, treating a variety of central nervous system related disorders. The system can also be used as bi-directional communication tool between brain and computer. In addition to the development of Brain Interchange as a platform for CNS related therapy discovery and brain-machine interface studies, he detailed that his company, CorTec provides the innovative technology that enables building the individual components of Brain Interchange to others. Especially the technology for producing laser-micromachined neural electrodes that permit high production precision while using only traditional implant materials such as medical grade silicone elastomers and PtIr noble metal foil. This technology can be applied to a wide range of applications, also in the field of spinal cord stimulation and peripheral nerve interfacing, in preclinical as well as in clinical studies. Another technology made available to researchers is a ceramic hermetic package for electronic implant circuits that permits a very high number of electrical feedthroughs (100 s) while providing excellent hermeticity was presented. Neuromodulation technology, including electronics, electrodes, and hermetic packaging have evolved significantly over the last few decades to meet clinical needs. Unfortunately, the OEMs that build most clinical technologies do not see a market in the research space. Further, the extremely high costs of validating new technologies means that progress in the fundamental area of device technology development has remained stagnant. This has led to a lack of availability of products that can meet the flexible and modular needs of researchers in BEM. Companies such as CorTec that maintain strong ties to the research community are core contributors to the ability to develop new bioelectronic technologies and therapies.

Robert Rennaker from University of Texas at Dallas presented his work on translational technologies to aid persons with neurological injuries. His team at UT Dallas has raised over $40 M in the past 7 years to develop technologies and therapies to treat neurological injuries. They have also participated in 5 clinical trials over the past 7 years. Notably, his team has designed, developed, and currently manufactures a miniature, wireless FDA class 3 implantable stimulator to enable the delivery of Targeted Plasticity Therapy. They have also developed an array of complementary technologies that can communicate with the implantable stimulator to deliver motor, sensory and cognitive rehabilitation. The devices and therapies that he presented work in conjunction with rehabilitative tasks to provide electrical stimulation to try to strengthen neural connections required to achieve full functional potential. Their device recently received FDA approval for clinical trials in PTSD, spinal cord injury, and stroke. Dr. Rennaker’s work is one of a handful of academic research groups that has brought an implantable device from preclinical research to clinical trials. This was achieved through rigorous preclinical research using multiple models to understand the mechanisms associated with the intended therapies (Dawson et al. 2016; Souza et al. 2019). This effort required securing a significant amount of funding, building a large collaborative research group, and establishing strategic partnerships with industry. Beyond the research efforts, they also had to learn to traverse the regulatory landscape which is atypical for academic groups. The level of effort required to achieve their goals is indicative of the very high bar required for successful translational of research in the field of bioelectronic medicine. Unfortunately, most academic research groups simply do not have the resources or the knowhow bring their research to this point. This points to a need to develop a research and development infrastructure to train, mentor, and equip research groups to be able to actually translate their work. Without this large-scale effort many potential therapies will remain in the research stage, and the benefits to the public of the many opportunities in BEM will be limited.

Chris Puelo from GE Research spoke about the research being done at GE in the area of therapeutic ultrasound based neuromodulation. He presented their latest results in studying and translating the using of the non-invasive neuromodulation technique to the clinic. Findings were presented from a recently completed clinical feasibility study was done in collaboration with the Feinstein Institutes for Medical Research under a joint collaboration with GE. Chris also discussed the data from their studies which helps to uncover some of the mechanisms underlying ultrasound-based nerve stimulation, and its application and utility across organ systems (Cotero et al. 2019) and diseases. In particular, the applications of ultrasound stimulation in the activation of the hepatoportal glucose sensor in models of type II diabetes were also covered in the presentation. Non-invasive neuromodulation has a much lower threshold for adoption compared to approaches that require surgery. Most currently available non-invasive devices use transcutaneous electrical stimulation. However, this approach does not allow easy targeting of specific fibers or locations because of current dispersion and dissipation through the intermediate tissues. Ultrasound has a long history of clinical application in diagnostic imaging, which has led to the development of advanced techniques for focused targeting of specific regions (Cotero et al. 2020). Although the exact mechanisms of neural activation due to ultrasound are not well known, studies like the work being done at GE show that there is a clear potential for therapeutic benefit, while helping to reveal some of the possible mechanisms.

## Frontiers in bioelectronics development

This session was chaired by Theo Zanos and Stephan Bickel from The Feinstein Institutes. Research at the forefront of BEM are driving the field forward. The speakers at the summit covered a wide range of related topics: development of biocompatible, electronic materials and their novel potential applications; the challenges and potential solutions when translating animal study results to humans; and new techniques to control sphincter muscles and to stimulate nerves such as the vagal nerve near their target organs.

Magnus Berggren from Linköping University gave an overview of a multitude of innovative projects and achievements using biocompatible, bioelectronic materials. Specifically, Dr. Berggren and his colleagues are using organic electronics that can conduct and process electronic charges, ions, and biomolecules that can be used in various device architectures for example by printing them in foils and patches. The potential use as sensors (e.g. electronic labels to monitor health status parameters), communicators, and actuators (e.g. electronic ion pump) is broad. Dr. Berggren presented several use cases including the BioComLab technology platform which combines various bioelectronic sensors (e.g., electronic skin patches) and actuators (e.g. drug delivery components) with communication technology to form an integrated system for future healthcare applications. While he presented use cases for the treatment of epilepsy and chronic pain, the potential for this platform is much wider and could possibly target neuronal disorders such as Parkinson’s disease, tremors, and non-neuronal disorders.

Kip Ludwig from University of Wisconsin, summarized important insights into the barriers and opportunities in the transition of findings from preclinical and translational research projects to the clinic. Important lessons can be learned from drug development and discovery, since most key factors associated with the large percentage of drugs failing to reach market approval are the same for many neuromodulation devices. As prior studies have outlined (Gupta 2011), the lack of predictability of animal models for humans, the lack of incorporation of dynamics in early-stage clinical trials to establish and confirm drug activity, as well as the lack of validated biomarkers for on and off-target effects, are common threads between both fields of drug and device development. To overcome these barriers, Dr. Ludwig proposed several strategies that already show promising results in published studies. One of the strategies focuses on cross species comparative anatomy, especially in large animal models, like the domestic pig, that could potentially capture variability across subjects (Settell et al. 2020). Another approach focused on isolating all possible sources of on and off-target effects, both neuronal and non-neuronal (Nicolai et al. 2020; Cheng et al. 2020). Development of better tools to understand neuronal and non-neuronal target engagement is also crucial. Finally, computational models that are functionally validated across species can assist in this difficult transition.

Larry Miller from The Feinstein Institutes presented how novel approaches to direct electrical stimulation of the vagus nerve and sphincter muscles can be used to control both constriction and relaxation of these important muscles in the gastro-intestinal tract. Sphincters control the passage of solids, liquids and gases through body orifices and are crucial for normal physiological functioning. As an additional important innovation, he proposed to stimulate peripheral branches of the vagus nerve close to their target organs, as opposed to the currently used stimulation of the cervical vagus nerve to increase specificity of the desired physiological effect and decrease side effects. Dr. Miller presented data from tunneling approaches and the use of endoscopic ultrasound approaches to guide needle electrodes to these peripheral nerve and ganglion targets in a porcine model. These techniques have the potential to treat a multitude of diseases including gastroesophageal reflux disease (GERD), diabetes, pulmonary and systemic hypertension and others.

The speakers and panel discussion outlined the constantly evolving landscape of BEM field, and how emerging challenges can become opportunities for innovation in multiple different complementary fields. The breadth and novelty of current cutting-edge tools and new findings reflects the interdisciplinary nature of the field and the combination of solid scientific foundations and creative thinking that can push the frontiers of BEM and deliver unique solutions to diagnose and treat a variety of diseases and conditions.

## Defining circuits

This session was chaired by Eric H. Chang from the Feinstein Institutes and Peder Olofsson from Karolinska Institute. Mapping and evaluating neural circuitry that can be therapeutically targeted in BEM is a major focus of various research groups across the globe. At the summit, recent developments in understanding the neural circuits that regulate physiological processes in immune, cardiovascular, and metabolic disorders were discussed with a specific focus on neural circuits interacting with the immune system in health and disease.

Saroj Mohanta’s from Institute for Cardiovascular Prevention, Ludwig-Maximilians-University presentation centered on his discovery of a neural atherosclerosis-brain-circuit (ABC) that connects atherosclerotic arteries with the CNS in atherosclerosis-prone ApoE deficient mice. Atherosclerotic lesions are a major cause of heart attacks and strokes, but whether neural signals play a role in the pathogenesis of atherosclerosis has been unknown. Using tissue clearing and light-sheet imaging of whole abdominal aorta tissues in advanced stages of atherosclerosis, Dr. Mohanta’s team found axon neogenesis in the artery adventitia and artery tertiary lymphoid organs (ATLOs) adjacent to plaques. Nerve tracing experiments of the ABC showed that afferent nerves entered the CNS central nervous system through dorsal root ganglia, and efferent nerves projected from the brainstem to the adventitia. Pharmacological or surgical perturbation of select components in the ABC changed the progression of atherosclerotic plaques. This research reveals a novel circuit that connects atherosclerotic plaques to the brain and suggests that altering nerve signals in this circuit may affect the course of atherosclerotic disease.

Daniela Carnevale from “Sapienza” University di Roma and IRCCS Neuromed discussed angiotensin II (Ang II)-induced hypertension in genetic mouse models and specific Ang II-responsive T-cells in the spleen. Dr. Carnevale showed that Ang II in the brain induces splenic sympathetic nerve activity, and that mice subjected to celiac ganglionectomy are protected from Ang II-induced induced hypertension. The work identifies an autonomic nervous system pathway involved in blood-pressure and hypertension control. Furthermore, placental growth factor (PIGF) in the spleen was required for the Ang II-induced hypertensive response in mice. In this circuit, Ang II activates neurons in the brain and efferent signals are propagated to the spleen through the vagus and splenic nerves, which promote the PIGF release in the spleen required for Ang II-dependent development of hypertension (Carnevale et al. 2016; Carnevale and Lembo 2021).

Silvia V. Conde from CEDOC, Nova Medical School presented the main findings of her research focused on the role of the carotid body (CB) in metabolism (Conde et al. 2020). The CB is a polymodal sensory organ capable of detecting glucose, leptin, and insulin in the blood. Abnormalities in CB and its sensory nerve, the carotid sinus nerve (CSN), have been associated with several metabolic disorders such as prediabetic insulin resistance and type II diabetes. Her research in preclinical models of diabetes and in human trials with hyperbaric oxygen therapy have demonstrated that CB-associated activity may be a therapeutic target for metabolic deregulation. Specifically, electrical neurostimulation using a kHz-frequency has been shown to block CSN activity, which Dr. Conde reports can be overactive in certain metabolic diseases. Dr. Conde’s work identified a neural signature in the CSN and sympathetic nerve activity and suggested that these structures could be a target for personalized bioelectronic therapeutics to treat metabolic disease (Conde et al. 2020; Sacramento et al. 2018).

## Clinical trial updates

This two-part session was chaired by Ona Bloom, Cynthia Aranow, and Ashesh Mehta from the Feinstein Institutes. The application of bioelectronic technology in the treatment of human disease is the ultimate goal of BEM. Since the discovery of the inflammatory reflex by Tracey and colleagues two decades ago (Borovikova et al. 2000; Tracey 2002), VNS has been increasingly explored in clinical trials to suppress excessive inflammation. In addition to reducing proinflammatory cytokines via the inflammatory reflex, a second mechanism by which VNS facilitates the resolution of inflammation is its effect on mediators of inflammation and resolution of inflammation, i.e. lipidomics (Mirakaj et al. 2014). A recent search of clinicaltrials.gov yielded 77 actively recruiting clinical trials of VNS, in diseases of chronic inflammation, as well as in stroke, depression, diabetes mellitus and others.

VNS has beneficial effects in preclinical animal models of both IBD and RA. Bruno Bonaz from University of Grenoble Alpes, INSERM and David Chernoff SetPoint Medical, discussed current developments. Both of these investigators focused on engaging the inflammatory reflex by direct VNS for treatment of chronic inflammatory conditions; Dr. Bonaz discussed the use of VNS to treat IBD and Dr. Chernoff discussed the use of VNS to treat RA. IBD is a chronic inflammatory disease affecting the intestine and is characterized by a remitting, relapsing course. Dr. Bonaz and colleagues have completed a 12 month open label clinical trial of VNS administered using a surgically implanted device in 9 participants with mild to moderately active IBD (Sinniger et al. 2020). Two of the 9 participants experienced no benefit and required initiation of pharmacologic treatment. The remaining 7 participants not only experienced symptomatic improvement, but were also found to have endoscopic improvement, biomarker improvement (fetal calprotectin) and decreased levels of circulating proinflammatory mediators. Positive effects were noted on vagus tone and HR-EEG. VNS was feasible, well tolerated and was not associated with any major side effects.

The effect of VNS on RA disease activity has been assessed in two trials. In a pivotal proof of concept study, Koopman and colleagues applied VNS to individuals with RA who were refractory to available pharmacological management, to evaluate the potential benefit of this approach (Koopman et al. 2016). In this open-label study, 17 patients received daily VNS via a surgically implanted stimulator. After 6 weeks of VNS, disease activity was significantly reduced. Furthermore, TNF production by LPS stimulated whole blood was attenuated from baseline. Discontinuation of VNS for 2 weeks led to an increase in both disease activity as well as stimulated TNF production, but reinitiating VNS resulted in reduced disease activity which has been sustained through a follow-up of at least 2 years. Only 2 participants required re-initiation of biologic therapy to control their disease. A second trial was recently completed which evaluated the effects of VNS for 12 weeks in 14 patients with long-standing RA and disease activity which was resistant to treatment to multiple pharmacologic agents (Genovese et al. 2020). Four participants underwent sham stimulation; 5 of 10 treatment refractory patients receiving VNS achieved a clinically meaningful reduction in their disease activity and two achieved remission. In addition to reduced production of proinflammatory cytokines from whole blood stimulated by LPS ex vivo, there were trends showing preservation of joint structure among study participants receiving VNS.

Beyond VNS, BEM has expanded in recent years to include the development of new biosensors and biocompatible materials, neuromodulation and smart devices, computer applications that improve communication between patients and healthcare providers, as well as computational methods to analyze big data. Journals such as Bioelectronic Medicine, Nature Machine Intelligence, Frontiers in Big Data and others, provide a new and specific platform to focus on studies in this area. Suraj Kapa from Mayo Clinic gave an overview of these aspects of bioelectronic medicine and many of the challenges faced by the field. He highlighted the enormous quantity of data obtained by biosensors and the need for artificial intelligence (AI) and machine learning to develop algorithms to best understand the information captured. Prediction of atrial fibrillation (a heart arrhythmia) using machine intelligence was one concrete example of the advantage achieved by supplementing clinical intelligence with machine intelligence.

Together, these studies represent a novel approach to the control of inflammatory disease and the growing application of bioelectronic medicine. Many patients do not respond or lose their response to currently available treatments. Pharmacologic therapies used to reduce inflammation are associated with numerous side effects including increased susceptibility to infection. A safe and non-immunosuppressive modality is an extremely attractive approach for control of inflammatory features of disease and additional studies evaluating this promising intervention and its potential mechanisms in these and other diseases are warranted.

Dr. Douglas Weber described his experience with restoration of sensory and motor function using prosthetics. This technology relies critically communication between neural tissue and prosthetic devices. Examples of these include intracerebrally implanted arrays such as Utah arrays (Maynard et al. 1997) and wearable devices such peripheral muscle recording “sleeve” arrays (Liu et al. 2021). While chronic intracerebrally implanted microelectrode arrays have been shown to provide neurophysiological data with sufficient resolution and decoding accuracy to enable intended control of robotic arms (Collinger et al. 2013), the requirement for brain surgery is an impediment to more widespread use. An alternative noninvasive method of brain machine interface relies upon residual electromyographic activity in paralyzed patients. High resolution electromyographic “sleeve” arrays can reveal spatiotemporal patterns that may be used to decode intended movements. Application of motor unit decomposition analytical methods, enables a finer grained decoding that can detect single motor unit activity that can be applied to individual digit movements (Holobar and Farina 2014). This represents an important potential noninvasive advance to help restore function in paralyzed individuals using brain machine interface.

Dr. Daniel Yoshor described efforts to restore vision with intracerebral stimulating arrays (Beauchamp et al. 2020). The clinical circumstance of implanted subdural stimulating/recording electrodes in epilepsy patients permits the opportunity to test the effects of stimulating the brain to create percepts. Electrical stimulation of early visual areas reliably produce phosphenes, perceptions of spots of light at predicted and reliable locations in the visual field (Murphey et al. 2009). While stimulation of individual sites produces spots, it has not been possible to restore holistic visual patterns with static stimulation of multiple sites. On the other hand, dynamic current steering, by sweeping electrical stimulation across the cortex in a manner that leverages the retinotopic mapping of the brain, produces more coherent and useful visual forms. Using this insight gained from sighted patients with epilepsy, this dynamic stimulation mapping method has been applied to blind subjects whose visual cortex is implanted with the Neuropace device, one that may be used to both record from and stimulate the brain. Using the Neuropace stimulating device in conjunction with the Orion goggle visual transducing device, patterns of stimulation are produced in the visual cortex of blind patients. With visual cortical stimulation, blind subjects are able to localize squares of light, navigate in a lighted environment and show some evidence of pattern discrimination. This represents an important advance in brain machine interface and restoration of vision in the blind.

## Summary and future directions

Preclinical research and clinical trials continue to provide new insights into the scope of the broad applications of BEM. There are important ongoing advances in the “building blocks” of BEM, including biomaterials, organic electronics, neural interfaces, and implantable devices that will be key to the development of new BEM discoveries. These building blocks are being studied in preclinical settings for both basic scientific discovery and to determine their utility in chronic disease models. Neural circuits with roles in the broad spectrum of diseases are constantly being identified and targeted by bioelectronics for therapeutic benefit in pre-clinical settings (Tsaava et al. 2020). One of the major active areas for development is the ability to perform chronic stimulation in rodent models (Wright et al. 2019; Mughrabi et al. 2021), allowing the leveraging of the myriad of disease models into BEM therapy discovery. Understanding the anatomical differences among rodents, pigs and humans will help to refine current neuromodulation approaches, establish a pathway for translation from rodents to larger models, and drive future successful clinical trials. Interactions among preclinical scientists, clinicians and industry are key for taking rigorous pre-clinical research to clinical explorations. The ongoing utilization of VNS in clinical settings of RA and IBD continues to generate evidence for efficacy and safety in BEM. A growing number of patients with these and other disorders and conditions have started to receive significant benefit from VNS and other new BEM therapeutic approaches (Qureshi et al. 2020). VNS has also been successfully used in approaches for stroke rehabilitation. There are examples of successful use of ultrasound in treating inflammatory and metabolic conditions in preclinical and clinical settings (Cotero et al. 2019; Huerta et al. 2021). Artificial intelligence (AI) is becoming a vital component of BEM with implications in neuroscience, cardiology, and many other clinical fields by allowing distillation and dissemination of information contained in neural signals and experimental data. There are key advances in bioelectronic limbs and prostheses, wearable and injectable sensors in paralysis and spinal cord injury, and brain stimulation for the blind including visual cortical prosthetics. The BEM community is growing, united by the common goal to help patients alleviate or cure their diseases. The BEM field is evolving and alongside the emerging new opportunities there are ever-present challenges. They are related to technological needs in devices and neural interfaces, translatability of neuromodulation regimens from pre-clinical to clinical settings in a specific disease context, ethical considerations, regulatory device approval and other aspects. These challenges drive further progress.

## Data Availability

Not applicable.

## Abbreviations

ABC: Atherosclerosis-brain-circuit
AI: Artificial intelligence
ANG II: Angiotensin II
ApoE: Apolipoprotein E
ATLO: Artery tertiary lymphoid organs
BEM: Bioelectronic medicine
CB: Carotid body
CNS: Central nervous system
CSN: Carotid sinus nerve
DBS: Deep brain stimulation
EEG: Electroencephalogram
FDA: Food and drug administration
GE: General Electric
GERD: Gastroesophageal reflux disease
GI: Gastrointestinal
Gmbh: Gesellschaft mit beschränkter Haftung
HR: Heart rate
IBD: Inflammatory bowel disease
INSERM: Institut national de la santé et de la recherche médicale
LPS: Lipopolysaccharide
NIH: National Institutes of Health
OEM: Original equipment manufacturer
PIGF: Placental growth factor
PtIr: Platinum Iridium
PTSD: Post traumatic stress disorder
RA: Rheumatoid arthritis
SNR: Signal to noise ratio
SPARC: Stimulating Peripheral Activity to Relieve Conditions
TNF: Tumor necrosis factor
VNS: Vagus nerve stimulation
